# Cannabis and Older Persons: Charting the Course From the Clinic to the Dispensary

**DOI:** 10.1093/geroni/igaf122.1966

**Published:** 2025-12-31

**Authors:** Brian Kaskie

## Abstract

Since 2020, the Cannabis and Older Persons symposium has featured panelists representing distinct academic disciplines offering unique perspectives about cannabis and aging. This year our panel features the latest work from individuals trained in clinical psychology, health services and policy, psychiatry, and public health. Erin Bonar and Erica Solway rely on the National Poll on Healthy Aging to discuss their latest findings about older adults’ attitudes and use of cannabis. Madeline Meier presents the DNA-methylation profiles of long-term cannabis users participating in the longitudinal Dunedin Study, a five-decade long study based in New Zealand. Ofir Livne presents differences in detailed cannabis use patterns and Delta-9 THC intake between older adults and younger age groups, and associations with cannabis use disorder. Cyril D’Souza considers how cannabis use corresponds with presentation of psychiatric symptoms among older persons. Divya Bhagianadh explores linkages between location of Medical Cannabis Dispensaries (MCDs) across 12 states with comprehensive medical cannabis programs and socioeconomic, and health characteristics of the neighborhood in which they are located. Together, these studies reflect how scientific understanding of cannabis use among older persons has been informed by multiple disciplinary perspectives, and how researchers ask and answer unique questions about a common topic with varied methodological approaches. This symposium offers researchers a state-of-the-science cannabis use among older adults.

